# Editorial: Serotonin and Memory

**DOI:** 10.3389/fphar.2016.00008

**Published:** 2016-02-01

**Authors:** Alfredo Meneses, Antonella Gasbarri

**Affiliations:** ^1^Departamento de Farmacobiología, Centro de Investigación y de Estudios Avanzados del Instituto Politécnico NacionalMexico City, Mexico; ^2^Department of Applied Clinical and Biotechnologic Sciences, University of L'AquilaL'Aquila, Italy

**Keywords:** serotonin, neural markers, therapeutic targets, memory, short-term, memory, long-term, memory disorders

Several neurotransmission systems have been involved in function and dysfunctional memory (e.g., Myhrer, 2003; Decker and McGaugh, 2004; Reis et al., 2009; Cassel, 2010; Rodríguez et al., 2012; Komal and Nashmi, 2015), including serotonin (5-hydroxytryptamine, 5-HT), which accounts with multiple neural markers (receptors, transporter; e.g., Hannon and Hoyer, 2008; Saulin et al., 2012; Seyedabadi et al., 2014; McCorvy and Roth, 2015). Indeed, the 5-HT system can be manipulated in multiple ways with pharmacological tools and possesses well characterized downstream signaling in mammals' species (e.g., Marin et al., 2012; McCorvy and Roth, 2015). Emergent evidence indicates that this monoamine system might be a therapeutic target and neural marker regarding function and dysfunctional memory. This issue presents recent advances including the role of 5-HT_2A_ and 5-HT_1A_ receptors in the medial prefrontal cortex during recognition memory (Morici et al.). Hippocampal 5-HT_1A_ receptors and spatial and memory is revised by Glikmann-Johnston et al. Ochoa et al. report that post-training serotonergic depletions of the basolateral amygdala did not disrupt discrimination, retention or reversal learning; suggesting that this serotonergic activity is not required for formation and flexible adjustment of new stimulus-reward associations when the strategy to efficiently solve the task has already been learned. Hernández-Pérez et al. report that serotonin reduction in the supramammillary nucleus alters place learning and concomitant hippocampal, septal, and supramammillar theta activity in spatial memory. Zhang and Stackman review progress in the 5-HT_2A_ receptor distribution, signaling, polymerization, and allosteric modulation; as well as functions in learning and memory, hallucination and spatial cognition, and mental disorders. Pereira et al. show us that 5-HT_6_ receptor agonism facilitates emotional learning and involves prefrontal cortex and hippocampal signaling. Serotonergic transporter function is reported by Sivamaruthi et al. demonstrating that cronobacter sakazakii infection alters serotonin transporter and improved fear memory retention. Stiedl et al. discuss the role of the serotonin receptor subtypes 5-HT_1A_ and 5-HT_7_ and their interaction in emotional learning and memory; including the role of these receptors and their interplay at the molecular, neurochemical, and behavioral level. The potential involvement of serotonergic neural markers with respect to memory is reviewed by Meneses.

Special mention and thanks to the expert work of the referees, who made professional and careful reviews that improved the papers in this topic.

## Author contributions

BG support as referee in several papers. AM was editor.

### Conflict of interest statement

The authors declare that the research was conducted in the absence of any commercial or financial relationships that could be construed as a potential conflict of interest.
