# Corrigendum: Editorial: Serotonin and Memory

**DOI:** 10.3389/fphar.2016.00036

**Published:** 2016-02-12

**Authors:** Alfredo Meneses, Antonella Gasbarri

**Affiliations:** ^1^Departamento de Farmacobiología, Centro de Investigación y de Estudios Avanzados del Instituto Politécnico NacionalMexico City, Mexico; ^2^Department of Applied Clinical and Biotechnologic Sciences, University of L’AquilaL’Aquila, Italy

**Keywords:** serotonin, neural markers, therapeutic targets, memory, short-term, memory, long-term, memory disorders

Due to an oversight, the name of Antonella Gasbarri in the Editorial article was reported as B. Gasbarri, which also rendered the citation of the Editorial article incorrect. This error does not change the scientific conclusions of the article in any way.

The original article was updated.

## Author contributions

Both authors listed have made substantial, direct and intellectual contribution to the work, and approved it for publication.

### Conflict of interest statement

The authors declare that the research was conducted in the absence of any commercial or financial relationships that could be construed as a potential conflict of interest.

